# MicroRNA-27a Induces Mesangial Cell Injury by Targeting of PPARγ, and its In Vivo Knockdown Prevents Progression of Diabetic Nephropathy

**DOI:** 10.1038/srep26072

**Published:** 2016-05-17

**Authors:** Lina Wu, Qingzhu Wang, Feng Guo, Xiaojun Ma, Hongfei Ji, Fei Liu, Yanyan Zhao, Guijun Qin

**Affiliations:** 1 Division of Endocrinology, Department of Internal Medicine, The First Affiliated Hospital of Zhengzhou University, Zhengzhou, 450052, China; 2 Institute of Clinical Medicine, The First Affiliated Hospital of Zhengzhou University, Zhengzhou, 450052, China

## Abstract

MicroRNAs play important roles in the pathogenesis of diabetic nephropathy (DN). In this study, we found that high glucose upregulated miR-27a expression in cultured glomerular mesangial cells and in the kidney glomeruli of streptozotocin (STZ)-induced diabetic rats. miR-27a knockdown prevented high glucose-induced mesangial cell proliferation and also blocked the upregulation of extracellular matrix (ECM)-associated profibrotic genes. Reduction of cell proliferation and profibrotic gene expression by a miR-27a inhibitor depended upon the expression of peroxisome proliferator-activated receptor γ (PPARγ). Further studies showed that miR-27a negatively regulated PPARγ expression by binding to the 3′-untranslated region of rat PPARγ. An antisense oligonucleotide specific to miR-27a (antagomir-27a) significantly reduced renal miR-27a expression in STZ-induced diabetic rats and significantly increased PPARγ levels. Antagomir-27a also reduced kidney ECM accumulation and proteinuria in STZ-induced diabetic rats. These findings suggest that specific reduction of renal miR-27a decreases renal fibrosis, which may be explained in part by its regulation of PPARγ, and that targeting miR-27a may represent a novel therapeutic approach for DN.

Diabetic nephropathy (DN) is a major microvascular complication of diabetes, and is the leading cause of end-stage renal disease. The key pathological hallmarks of DN are mesangial cell (MC) proliferation and an accumulation of extracellular matrix (ECM) proteins such as collagens and fibronectin. These processes are driven largely by the cytokine TGF-β1^1^,^2^,^3^. Despite substantial progress made in recent decades, our understanding of the underlying disease mechanisms is incomplete, and there is a need for identification of additional biomarkers and novel targets in order to improve DN treatment options.

MicroRNAs (miRNAs) are endogenously expressed, small noncoding RNAs that negatively regulate gene expression by binding to the 3′-untranslated region (3′-UTR) of target mRNAs^4^,^5^,^6^. miRNAs play important roles in cell growth, differentiation, proliferation, apoptosis, and cell death, and contribute to the pathogenesis of many human diseases, including cancer, diabetes, and diabetic complications such as DN in addition to other potential diseases^7^,^8^,^9^. The miR-27 gene family (miR-23a, 27a, and the 24-2 cluster) contributes to the regulation of cell cycle progression, proliferation, and hypertrophy^10^. Recently, Nielsen *et al*.^11^ demonstrated that 12 human miRNAs, including miR-27a, were upregulated in the serum of patients with type 1 diabetes. miR-27a expression is also upregulated in both metabolic syndrome and type 2 diabetes, and there is a strong positive correlation between miR-27a and fasting glucose levels^12^. In addition, miR-27a is upregulated in adipose tissue in a spontaneous rat model of type 2 diabetes mellitus, and its expression is increased in adipocytes in response to hyperglycaemia^13^. Collectively, these results suggest a role for miR-27a in the pathophysiology of type 1 and 2 diabetes. Moreover, miR-27a is primarily expressed in adipose tissue, lung, heart, and kidney^14^, suggesting that it may play an important role in renal function. However, a role of miR-27a in DN has not been reported to date.

Peroxisome proliferator-activated receptor γ (PPARγ), a potential target of miR-27a^14^, plays a critical role in ameliorating the course of DN^15^,^16^. The 3′-UTR of PPARγ contains a binding site for miR-27a, and miR-27a was reported to directly bind to PPARγ mRNA and regulate adipocyte^14^ and human pulmonary artery endothelial cell^17^ differentiation. PPARγ activation prevents high glucose (HG)-induced increases in TGF-β1 expression^18^, modulates renal MC proliferation and differentiation^19^, and inhibits HG- or TGF-β1-stimulated synthesis of type I collagen^20^. However, it is unclear whether there is a functional interaction between miR-27a and PPARγ with regard to MC proliferation or ECM deposition in DN.

In the current study, we found that miR-27a was upregulated in cultured glomerular MCs and in kidney glomeruli from streptozotocin (STZ)-induced diabetic rats. We also provide strong experimental evidence that miR-27a negatively regulates the expression of PPARγ through the PPARγ 3′-UTR. Moreover, our results confirmed that miR-27a downregulation increases PPARγ expression, reduces MC proliferation and ECM accumulation, and ameliorates proteinuria. Thus, our results establish a critical role for miR-27a in the pathophysiology of DN.

## Research Design and Methods

### Reagents and antibodies

Streptozotocin (STZ) was supplied by Sigma (Saint Louis, MO, USA). Lipofectamine2000 was supplied by Invitrogen (Grand Island, USA). The miR-27a mimic, a miRNA negative control (NC mimic), miR-27a inhibitor, and miRNA inhibitor negative control (NC inhibitor) were designed and synthesized by GenePharma (Shanghai, PRC). Antagomir-27a, a specific PPARγ small interfering RNA (siRNA), as well as a scrambled RNA (scRNA) were designed and synthesized by RiboBio (Guangzhou, PRC). The following antibodies were used: rabbit anti-rat polyclonal antibodies including anti-PPARγ (Santa Cruz Biotechnology, CA), anti-TGF-β1 and anti-PAI-1 (Cell Signaling Technology, USA), anti-GAPDH (CWBIO, Beijing, PRC), anti-collagen IV and anti-fibronectin (Bioss, Beijing, PRC).

### Cell culture and transfection

The rat MC line (HBZY-1) was purchased from the Cell Culture Centre of the Institute of Biomedicine and Health (Guangzhou, PRC) and cultured as previously described^21^. Briefly, MCs were maintained at 37 °C in Dulbecco’s modified Eagle’s medium (Invitrogen, USA) supplemented with 10% foetal bovine serum (Gibco, USA) in an atmosphere containing 5% CO_2_. Cells were treated with the indicated concentrations of glucose. Twenty-four hours prior to transfection, 2.0 × 10^5^ MCs were plated into a 6-well plate. MCs were transfected using Lipofectamine2000 with final concentrations of 50 nM miR-27a mimic or miR-27a inhibitor and/or PPARγ siRNA. The medium was replaced with fresh culture medium 4–6 h after transfection, and MCs were harvested for subsequent studies at 48 or 72 h after transfection.

### Animal experiments

Eight-week-old male Sprague–Dawley rats (220 ± 20 g) were purchased from the Experimental Animal Centre of Henan Province. All animal studies were approved by the Animal Care and Use Committee of the First Affiliated Hospital of Zhengzhou University and fully complied with the University guidelines for the care and use of laboratory animals. Diabetes was induced by a single intraperitoneal injection of 60 mg/kg STZ after fasting for 12 h. Blood glucose was measured to validate the induction of diabetes (>16.7 mmol/L) 72 h after STZ injection. Rats that received an injection of diluent buffer alone served as the normal control group (control, n = 5). Antagomir-27a or miR-27a mismatch mutations (NC antagomir) were administered to diabetic rats (n = 5, respectively) by intravenous injection at doses of 100 nM in 0.2 mL twice a week for 8 weeks. Body weight and blood glucose levels were monitored, and 24-h urine samples from each rat were collected every week. The rats were anesthetized with pentobarbital sodium (50 mg/kg body weight), and kidneys were harvested 24 h after the final injection. The kidney cortex was removed as described^22^, snap-frozen, and stored at −80 °C. Kidney glomeruli were isolated from cortical tissues by following a sequential sieving method^22^.

### Quantitative real time (qRT)-PCR for miR-27a

miRNAs from cultured MCs and kidney glomeruli were isolated using the E.Z.N.A. miRNA Kit (Sigma, USA). cDNA was synthesized using miRNA-specific reverse transcription primers and the TaqMan MicroRNA Reverse Transcription Kit (Applied Biosystems, USA). miRNA-27a expression was quantified using miRNA-specific PCR primers and probe and the TaqMan Gene Expression Master Mix (Applied Biosystems, USA) on an Applied Biosystems 7500 Fast Sequence Detection System (Applied Biosystems). U6 small nuclear RNA (snRNA) served as an internal control and was amplified with forward (5′-ATTGGAACGATACAGAGAAGATT-3′) and reverse (5′-GGAACGCTTCACGAATTTG-3′) primers, and detected with a probe (5′-TGCGCAAGGATGACACGCA-3′). All primers and probes were obtained from GenePharma (Shanghai, PRC). Each sample was run in triplicate, and each experiment was repeated at least 3 times. Relative expression of miR-27a was analysed using the 2^−ΔΔCT^ method^23^.

### Luciferase assays and site-directed mutagenesis

For luciferase assays, rat kidney MCs were cotransfected with the pEZX-MT05 vector with a PPARγ-3′-UTR (GeneCopoeia RmiT049429-MT05) or a mut-PPARγ-3′-UTR (GeneCopoeia CS-RmiT049429-MT05), the pEXZ-MR03 vector encoding the miR-27a mimic (GeneCopoeia RmiR6129-MR03), and the pEXZ-AM03 vector encoding the miR-27a inhibitor (GeneCopoeia RmiR-AN0359-AM03), using the EndoFectin Lenti Transfection Reagent (Cat.#: EFL1001-01, GeneCopoeia, Rockville, MD, USA). Following a 48-h transfection period, Gaussia luciferase (GLuc) and secreted alkaline phosphatase (SEAP) activities were measured using a Secrete-Pair Dual Luminescence Assay Kit (Catalogue # SPDA-D010, GeneCopoeia, Rockville, MD, USA) and a luminometer. SEAP activity was measured as an internal control. For each transfection, luciferase activities were averaged from 3 replicates.

### RNA extraction and qRT-PCR

Total RNA was extracted from cultured MCs and kidney glomeruli using the Trizol reagent (TaKaRa Bio, Japan). First-strand cDNAs were synthesized using a cDNA Synthesis Kit (TOYOBO, Japan) and then quantified by real-time PCR with the KOD SYBR Green qPCR Mix kit (TOYOBO, Japan) and appropriate primers. Reactions were performed on the ABI 7500FAST System (Foster City, CA). Relative gene expression levels were normalized to GAPDH expression. Primers were obtained from Sangon Biotech Co., Ltd. (Shanghai, PRC), and all primer information is available upon request.

### Western blot analysis

Total protein was extracted from MCs and kidney glomeruli with a protein extraction reagent (Thermo, USA). Western blot analysis was performed as described previously^24^. Membranes were exposed to a rabbit anti-PPARγ antibody (diluted 1:100), or to a rabbit anti-TGF-β1 or rabbit anti-PAI-1 antibody (diluted 1:1000). After incubation with the HRP-conjugated secondary antibody, blots were visualized with the Pierce ECL Western Blotting Substrate (Thermo Fisher Scientific, USA). Relative proteins expression levels were normalized to GAPDH levels.

### Immunofluorescence microscopy

MCs were seeded onto chamber slides and transfected with the miR-27a mimic, the miR-27a inhibitor, or a negative control miRNA. At 48 h after transfection, cells were fixed in 4% paraformaldehyde solution for 20 min, permeabilized with 0.25% Triton X-100 for 15 min, and blocked with 1% BSA for 30 min at room temperature. After overnight incubation at 4 °C with a 1:50-diluted anti-PPARγ antibody, MCs were exposed to a Cy3-labelled secondary antibody (diluted 1:500) for 1 h at room temperature, followed by DAPI staining for 2 min. The cellular distribution and localization of target proteins was examined under a fluorescent microscope (IX71; OLYMPUS, Japan).

### Growth inhibition test

The Cell Counting Kit-8 (CCK-8) was employed to analyse cell proliferation, as described previously^25^. MCs were plated at a density of 5,000 cells/well in 96-well plates, and subsequently transfected with the miR-27a inhibitor, PPARγ siRNA, or negative controls at a final concentration of 50 nM. At 48 h after transfection, cell proliferation was measured with the CCK-8 Kit (BestBio, Shanghai, PRC). Each assay was performed with 6 replicates in 3 independent experiments.

### Immunohistochemistry

Paraffin sections of rat kidneys were prepared by a conventional method and treated as we described previously^26^. The anti-collagen IV and anti-fibronectin antibodies were diluted 1:100 and 1:200, respectively, for immunohistochemical staining experiments.

### Light and electron microscopy

Kidney pathology in periodic acid-Schiff (PAS) and haematoxylin and eosin staining sections was examined by light microscopy. Glomerular area and mesangial expansion index were quantified using Image-Pro Plus 6.0 software. The renal cortex of kidneys was dissected into small pieces (1 mm^3^) and treated as we described previously^26^. The ultrastructure of renal cortices was examined with an H-7500 transmission electron microscope (HITACHI, Japan).

### Statistics

All data were analysed with SPSS 18.0 software (IBM, Endicott, NY, USA) and are presented as mean ± S.E. Comparisons between more than 2 groups were assessed with one-way analysis of variance, followed by a Bonferroni test, and *P* values < 0.05 were considered statistically significant.

## Results

### Upregulation of miR-27a expression under hyperglycaemic conditions both *in vitro* and *in vivo*

**Figure 1:**
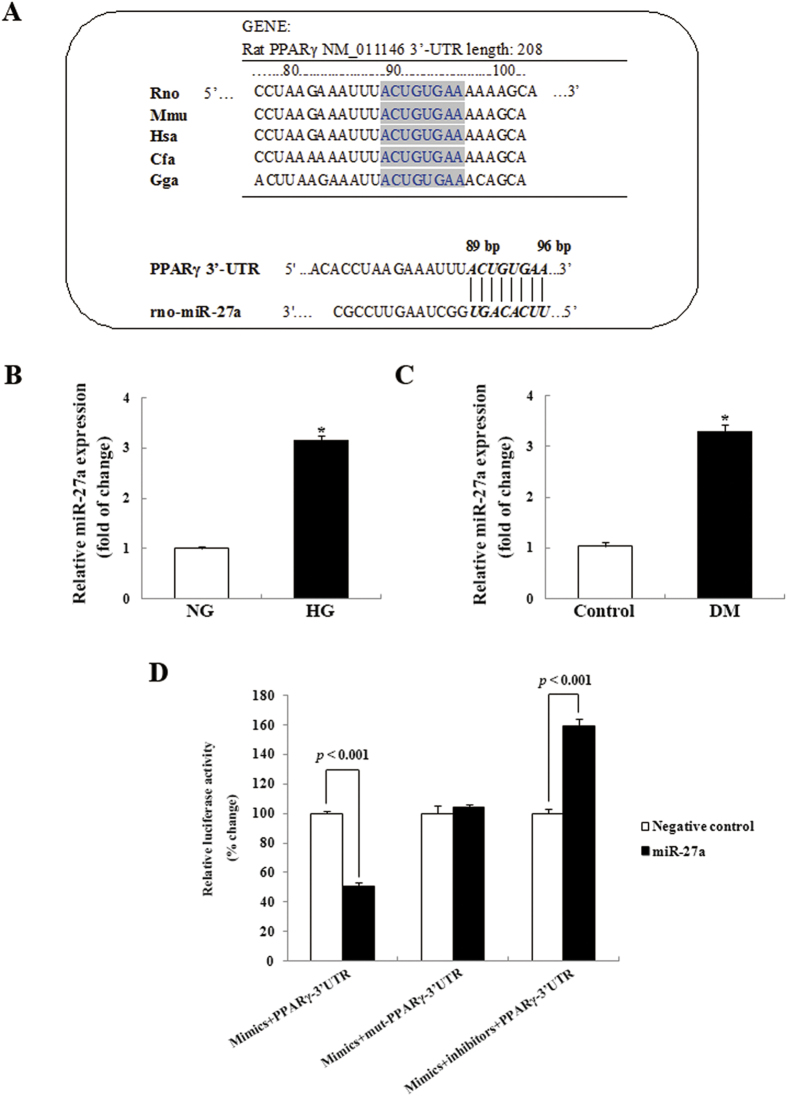
Expression of miR-27a is upregulated by high glucose, and targets the PPARγ gene. (**A**) *Upper panel*, conservation of the miR-27a binding site in the 3′-UTR of mammalian PPARγ genes. The miR-27a seed sequence is shown in the gray box. *Lower panel*, schematic illustration of miR-27a pairing with the rat PPARγ 3′-UTR. *Rno, Rattus norvegicus; Mmu, Mus musculus; Hsa, Homo sapiens; Cfa, Canis lupus familiaris; Gga, Gallus gallus domesticus; rno -miR-27a, Rattus norvegicus- miR-27a.* (**B**) Real-time qPCR analysis showing miR-27a expression in rat kidney mesangial cells (MCs) treated with high glucose (HG, 25 mM) as compared with MCs treated with normal glucose (NG, 5.6 mM). miR-27a levels were normalized to U6 snRNA expression. Data are shown as mean ± S.E. (n = 3). (**C**) Real-time qPCR analysis showing miR-27a expression in kidney glomeruli from streptozotocin-induced diabetic rats (DM) and normal control rats. miR-27a levels were normalized to U6 snRNA expression. Data are shown as mean ± S.E. (n = 5). *p < 0.05 vs. NG; *p < 0.05 vs. control. (**D**) Rat kidney MCs were co-transfected with the pEZX-MT05 vector with a PPARγ-3′-UTR or mut-PPARγ-3′-UTR, and the indicated miR-27a mimic, inhibitor, or negative control. Following a 48-h transfection period, Gaussia luciferase (GLuc) and secreted alkaline phosphatase (SEAP) activities were measured. Each bar represents GLuc luciferase activity values normalized to those for SEAP activity, and the normalized values were expressed relative to MCs treated with the negative control miRNA. The results shown were obtained from 3 independent experiments.

*In vitro* and diabetic animal model studies have confirmed the beneficial role of PPARγ in diabetic kidney disease^27^,^28^. To examine potential miRNAs that regulate PPARγ expression, we utilized computational prediction programs (TargetScan, PicTar, miRanda, and miRGen) to identify potential binding sites for miRNAs in the PPARγ-3′-UTR. This analysis indicated that miR-27a has a high probability of binding the 3′-UTR of PPARγ mRNA and that the putative miR-27a binding sites in the PPARγ-3′-UTR are highly conserved between several mammals, such as humans, mice, rats, chickens, and dogs (Fig. 1A). Furthermore, miR-27a is highly expressed in the kidneys^14^, and is also upregulated in patients with type 1 or 2 diabetes^11^,^12^. Thus, we focused on miR-27a in our experimental models, both *in vitro* and *in vivo*. As shown in Fig. 1B, miR-27a expression was significantly upregulated in rat kidney MCs exposed to HG (25 mM glucose). To examine the potential relevance of miR-27a upregulation in DN, we utilized a STZ-induced diabetes model in rats. As shown in Fig. 1C, miR-27a expression was significantly increased in the kidney glomeruli of diabetic rats. Taken together, these findings suggest that increased miR-27a levels may explain previously reported reductions of PPARγ expression^20^,^29^, which may in turn contribute to DN pathology.

### PPARγ is a target of miR-27a

To verify that miR-27a binds directly to the 3′-UTR of PPARγ, we inserted the rat PPARγ-3′-UTR (with the normal miR-27a binding site sequence or a mut-PPARγ-3′-UTR) into the pEZX-MT05 vector, which was then transfected into rat kidney MCs. As shown in Fig. 1D, miR-27a suppressed activity of the wild type PPARγ-3′-UTR luciferase-reporter construct by 50% compared with cells cotransfected with a negative control. To determine the specificity of this result, MCs were cotransfected with the mutant PPARγ-3′-UTR vector and the miR-27a overexpression vector in MCs. In this case, no significant changes in luciferase activity were observed upon overexpression of miR-27a or the negative control. Moreover, when the miR-27a expression vector was cotransfected with the miR-27a-inhibitor vector and the wild type PPARγ-3′-UTR, the miR-27a inhibitor significantly counteracted miR-27a-mediated luciferase downregulation. These results clearly indicate that miR-27a directly specifically binds to the 3′-UTR of PPARγ and suppress PPARγ expression.

### Inhibition of miR-27a suppressed HG-induced downregulation of PPARγ in HG-treated MCs

**Figure 2:**
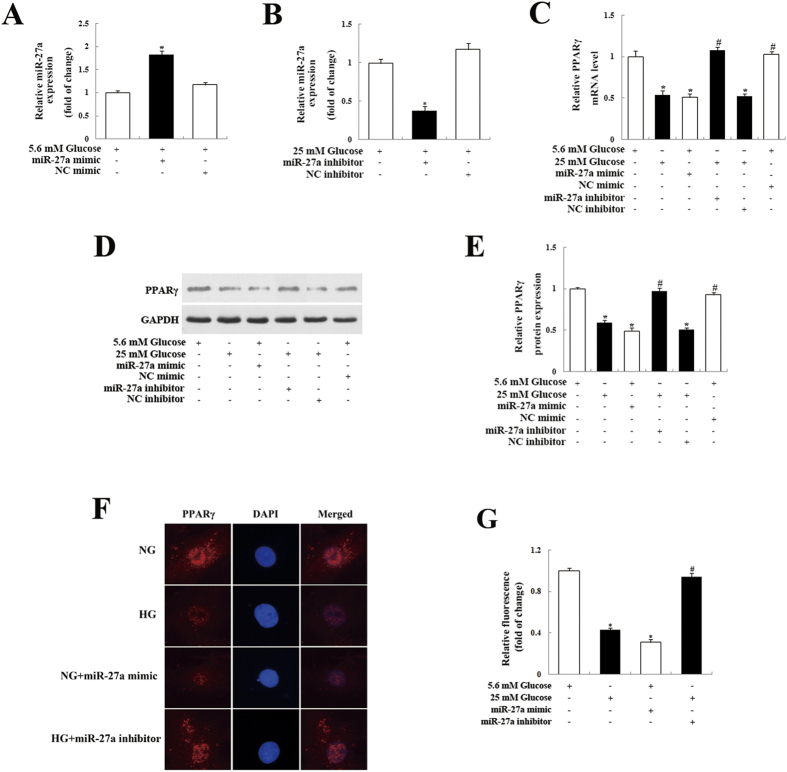
miR-27a negatively regulates PPARγ expression. Rat kidney mesangial cells were transfected with a miR-27a mimic under normal glucose (NG, 5.6 mM) conditions or a miR-27a inhibitor under high glucose (HG, 25 mM) conditions. (A, B) At 48 h after transfection, real-time qPCR analysis was employed to detect alterations in miR-27a levels. miR-27a levels were normalized to U6 snRNA expression. (**C**) Real-time qPCR analysis of PPARγ mRNA levels. (**D**) At 72 h after transfection, PPARγ protein levels were detected by western blot analysis. GAPDH served as a loading control. (**E**) Densitometric analysis of the PPARγ expression data shown in panel D. (**F**) Immunofluorescence detection of PPARγ. Original magnification, ×400. (**G**) Quantitative analysis based on PPARγ fluorescence intensities. Data are shown as mean ± S.E. (n = 3). *p < 0.05 vs. NG; ^*#*^*p* < 0.05 vs. HG.

To confirm the impact of miR-27a on PPARγ expression, MCs were transfected with a miR-27a mimic. As shown in Fig. 2, this lead to a clear increase in miR-27a levels in MCs (Fig. 2A) and a concomitant reduction in both PPARγ mRNA (Fig. 2C) and protein (Fig. 2D,E) levels. We next examined whether a miR-27a inhibitor would have the opposite effect. We found that HG treatment significantly decreased PPARγ mRNA (Fig. 2C) and protein (Fig. 2D,E) levels. Importantly, HG-induced downregulation of PPARγ was reversed when MCs were transfected with a miR-27a inhibitor (Fig. 2B–E). To further examine the effect of miR-27a on PPARγ, we performed immunofluorescence microscopy. Compared with normal glucose (NG)-treated MCs, PPARγ immunofluorescence was significantly reduced in MCs transfected with the miR-27a mimic and treated with HG (Fig. 2F,G). In contrast, suppression of miR-27a activity with the miR-27a inhibitor increased PPARγ immunofluorescence in HG-treated MCs (Fig. 2F,G). Taken together, these data indicate that miR-27a negatively regulates PPARγ expression.

### Inhibition of miR-27a attenuates MC proliferation and ECM accumulation

**Figure 3:**
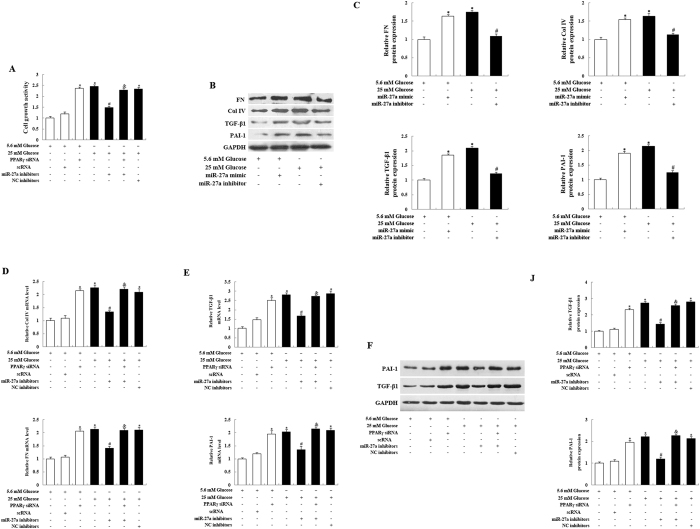
Downregulation of miR-27a with a miR-27a inhibitor prevented high glucose (HG)-induced mesangial cell (MC) proliferation and the expression of ECM-associated profibrotic genes via upregulation of PPARγ expression. Rat kidney MCs were transfected with PPARγ siRNA in the presence of NG levels (5.6 mM) conditions, or with a miR-27a inhibitor and/or PPARγ siRNA in the presence of HG (25 mM). (**A**) Cell proliferation rates were determined using the CCK-8 assay. The results shown were obtained in 3 independent experiments and represent the mean ± S.E. (B, C) Protein expression of collagen IV (Col IV), fibronectin (FN), TGF-β1 and PAI-1 was detected by western blot analysis. GAPDH served as a loading control. Data are shown as mean ± S.E. (n = 3). (**D**) Cellular expression of Col IV and FN was analysed by real-time qPCR, with normalization to GAPDH expression. Data are shown as mean ± S.E. (n = 3). (**E**) Real-time qPCR analysis of TGF-β1 and PAI-1 mRNA levels. Data are shown as mean ± S.E. (n = 3). (**F**) TGF-β1 and PAI-1 protein expression was detected by western blot analysis. GAPDH served as a loading control. (**J**) Densitometric analysis of the TGF-β1 and PAI-1 expression data shown in panel F. Data are shown as mean ± S.E. (n = 3). *p < 0.05 vs. 5.6 mM glucose; ^*#*^*p* < 0.05 vs. 25 mM glucose; ^*&*^*p* < 0.05 vs. 25 mM glucose + miR-27a inhibitor.

To determine the role of miR-27a in MC proliferation, we next examined cell viability. MC proliferation in the presence of HG was significantly increased compared with MCs cultured in NG conditions (Fig. 3A). In addition, the proliferation of miR-27a inhibitor-transfected MCs was significantly inhibited compared with HG-treated MCs and negative control cells. The result is in agreement with previous data showing that miR-27a downregulation suppresses cell growth *in vitro*^30^. In contrast, when HG-treated MCs were transfected with both the miR-27a inhibitor and PPARγ siRNA, the effect of miR-27a on cell growth was enhanced (Fig. 3A).

Because MCs contribute to the excessive accumulation of ECM proteins during DN, we next examined the effects of miR-27a on proteins relevant to this process. As shown in Fig. 3B,C, a marked increase in the protein levels of collagen IV, fibronectin, TGF-β1 and PAI-1 were observed in the HG group compared with the NG group. As expected, miR-27a inhibitor suppressed the HG-dependent upregulation the levels of these ECM-associated profibrotic genes. In addition, upregulation of miR-27a by miR-27a mimic increased the levels of these profibrotic genes, compared with NG group. These data indicate that mimic and inhibitor of miR-27a had the opposite effect on ECM accumulation. HG also increased mRNA expression of collagen IV, fibronectin, TGF-β1 and PAI-1 in MCs, as determined by qRT–PCR (Fig. 3D,E). However, the effect of HG was reversed by miR-27a inhibition. In contrast, the effect of miR-27a inhibitor was significantly decreased when HG-treated MCs were transfected with the miR-27a inhibitor and PPARγ siRNA (Fig. 3D–J). PPARγ activation is associated with reduced expression of ECM proteins and TGF-β1 in the glomeruli of STZ-induced diabetic rats^3^,^31^ and may directly attenuate diabetic glomerular disease by inhibiting PAI-1 expression^32^. Together with our findings indicated that alterations in miR-27a levels could regulate PPARγ levels (Fig. 2), these data demonstrate that HG-induced cell proliferation and ECM accumulation may be prevented by the inhibition of miR-27a, and that this is likely due to the consequent up-regulation of PPARγ.

### Knockdown of miR-27a protects renal function in STZ-induced diabetic rats and ameliorates DN progression *in vivo*

**Figure 4:**
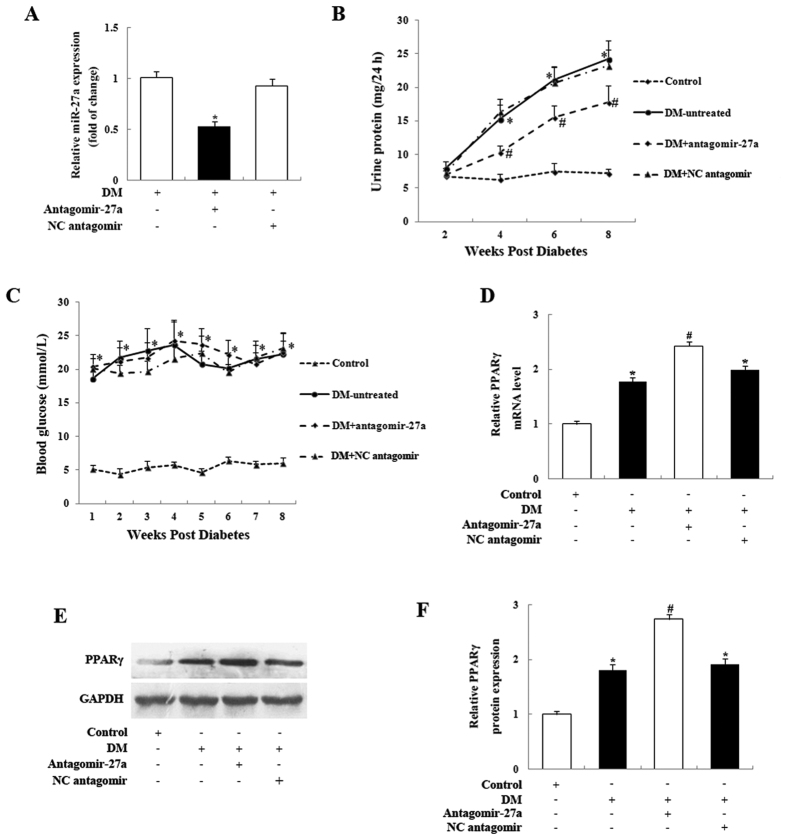
Inhibition of miR-27a with antagomir-27a reduces miR-27a expression, attenuates proteinuria, and increases PPARγ expression in STZ-induced diabetic rats. (**A**) Real-time qPCR data showing significantly decreased miR-27a expression in the kidney glomeruli of antagomir-27a-treated diabetic rats, as compared with untreated diabetic rats (DM) or negative control rats. miR-27a levels were normalized to U6 snRNA expression. Data are shown as mean ± S.E. (n = 5). (**B**) Injection of antagomir-27a led to improved proteinuria in STZ-induced diabetic rats (n = 5/group). (**C**) Blood glucose levels were not significantly affected by antagomir-27a treatment compared to untreated diabetic rats or negative control rats (n = 5/group). (**D**) Real-time qPCR analysis showed PPARγ expression levels were significantly upregulated in the kidney glomeruli of antagomir-27a-treated diabetic rats, compared with untreated diabetic rats or negative control rats. Data are shown as mean ± S.E. (n = 5). (**E**) Western blot analysis of PPARγ protein expression in the kidney glomeruli of antagomir-27a-treated diabetic rats, compared with untreated diabetic rats or negative control rats. (**F**) Densitometric analysis of the PPARγ expression data shown in panel E. Data are shown as mean ± S.E. (n = 5). *p < 0.05 vs. Control; ^*#*^*p* < 0.05 vs. DM.

Based on our *in vitro* data, we hypothesized that miR-27a inhibition *in vivo* might protect renal function in STZ-induced diabetic rats. Antagomirs, a novel class of chemically engineered oligonucleotides, are powerful functional inhibitors of miRNAs *in vivo*, and may represent a viable therapeutic strategy for silencing miRNAs in disease^33^,^34^,^35^. To test whether they would be effective in DN, we used a chemically modified antisense oligonucleotide (antagomir-27a) to knock down miR-27a expression *in vivo*. As shown in Fig. 4A, miR-27a expression in kidney glomeruli was significantly suppressed in antagomir-27a-treated diabetic rats, compared with untreated diabetic rats. NC antagomir did not affect miR-27a levels in diabetic rats. We also found that proteinuria was significantly increased in untreated diabetic rats compared with normal control rats (Fig. 4B). After 8 weeks of antagomir-27a treatment, however, we observed a marked improvement in proteinuria compared with untreated diabetic rats or negative control (NC antagomir) rats (Fig. 4B). However, blood glucose levels were not significantly affected by antagomir-27a (Fig. 4C). These results demonstrate that endogenous miR-27a levels are efficiently knocked down by antagomir-27a *in vivo*, and that this is associated with renoprotective effects in diabetic rats.

**Figure 5:**
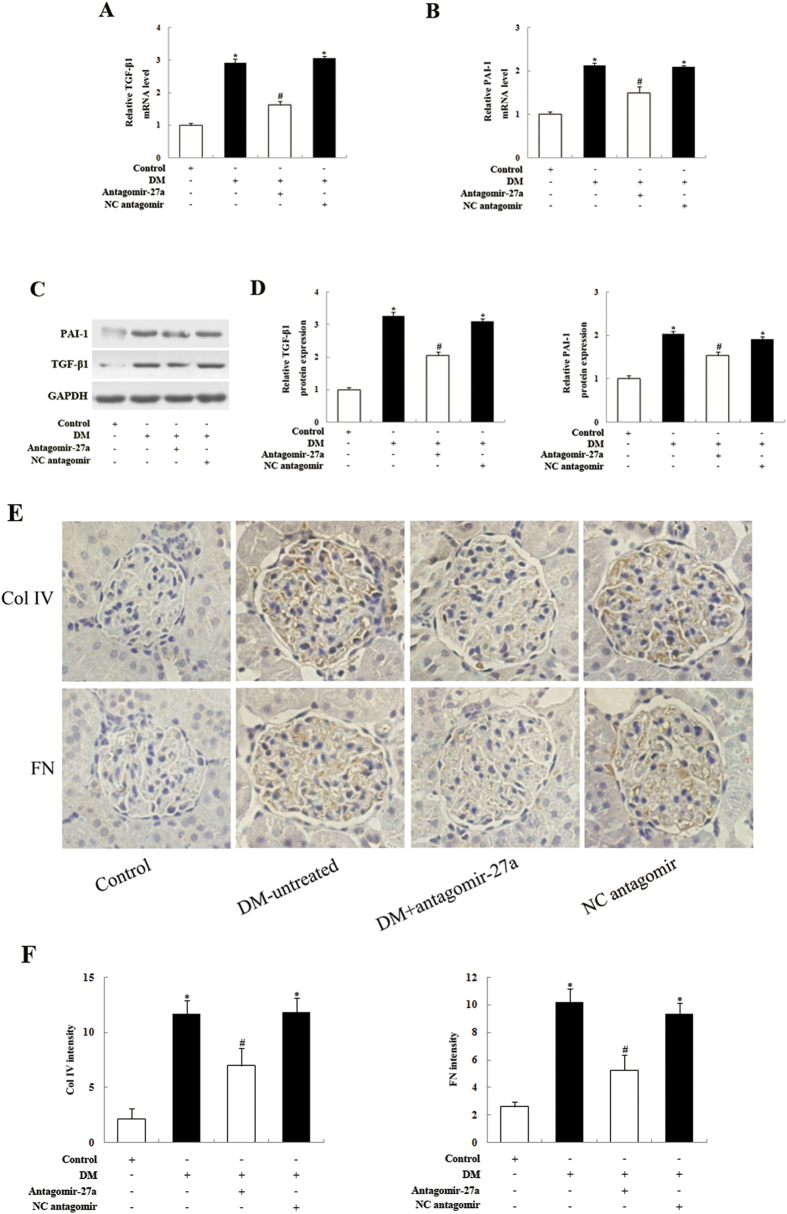
Inhibition of miR-27a with antagomir-27a reduces the expression of profibrotic genes and ECM proteins in the kidney glomeruli of STZ-induced diabetic rats. (A, B) Real-time qPCR analysis of TGF-β1 and PAI-1 expression in the kidney glomeruli of antagomir-27a-treated diabetic rats, compared with untreated diabetic rats or negative control rats. (**C**) Western blot analysis of TGF-β1 and PAI-1 protein expression in the kidney glomeruli of antagomir-27a-treated diabetic rats, compared with untreated diabetic rats or negative control rats. (**D**) Densitometric analysis of TGF-β1 and PAI-1 expression data shown in panel C. (**E**) Representative collagen IV (Col IV) and fibronectin (FN) immunohistochemistry staining of kidney sections in antagomir-27a-treated diabetic rats, compared with untreated diabetic rats or negative control rats. (**F**) Densitometry analysis of Col IV and FN. Data are shown as mean ± S.E. (n = 5). *p < 0.05 vs. Control; ^*#*^*p* < 0.05 vs. DM.

To determine the *in vivo* relevance of miR-27a knockdown on PPARγ expression in the kidneys of STZ-induced rats, we examined the expression of PPARγ mRNA and protein levels in the kidney glomeruli of antagomir-27a-treated rats. As shown in Fig. 4D–F, we confirmed that PPARγ mRNA and protein levels were significantly increased following injection of antagomir-27a. We also found that PPARγ mRNA and protein levels in the kidney glomeruli were increased in untreated diabetic rats compared with normal control rats, which is interesting given that the increase in PPARγ during tissue injury may limit inflammation and injury responses^32^. To further validate the renoprotective effect of antagomir-27a, we examined the levels of key ECM-associated profibrotic genes, such as TGF-β1, PAI-1, collagen IV, and fibronectin. In agreement with our *in vitro* results, the TGF-β1 and PAI-1 levels were reduced in kidney glomeruli samples from antagomir-27a-treated diabetic rats (Fig. 5A–D). Furthermore, immunohistochemistry revealed that collagen IV and fibronectin levels were significantly attenuated in diabetic rats injected with antagomir-27a (Fig. 5E,F). These results demonstrate that the inhibition of endogenous miR-27a by antagomir-27a and subsequent increase in PPARγ levels triggers the downregulation of several genes that play key profibrotic roles in diabetic rats.

**Figure 6:**
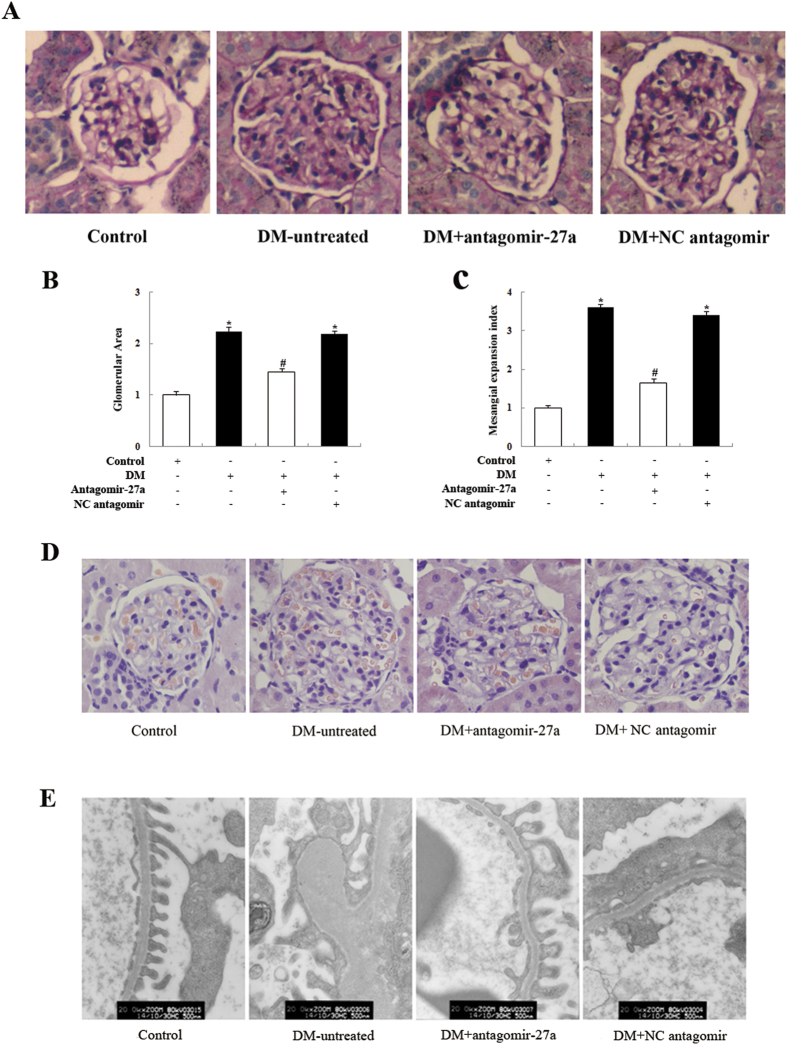
Effect of antagomir-27a on renal morphology in STZ-induced diabetic rats. (**A**) Representative PAS staining of kidney sections in antagomir-27a-treated diabetic rats, compared with untreated diabetic rats or negative control rats (×400). (**B**) Glomerular area in PAS-positive sections. (**C**) Mesangial expansion index defined by the ratio of mesangial area/glomerular tuft area in PAS-positive sections. Data are shown as mean ± S.E. *p < 0.05 vs. control; ^*#*^*p* < 0.05 vs. DM. (**D**) Haematoxylin and eosin staining of rat glomeruli (×400). (**E**) Ultrastructure of kidneys from rats treated as indicated, as visualized under a transmission electron microscope (×20,000).

To further validate our findings, we next examined the effect of antagomir-27a in the kidneys of treated rats by PAS and histopathological staining. This revealed an increased glomerular surface area, MC expansion, and thickened glomerular basement membranes in the kidneys of untreated diabetic rats. Conversely, renal pathology was ameliorated in diabetic rats injected with antagomir-27a (Fig. 6A–D). Moreover, electron microscopy revealed that podocyte morphology was severely compromised in both untreated diabetic rats and those treated with a negative control antagomir. In contrast, antagomir-27a ameliorated podocyte injury to some degree (Fig. 6E). These data further support the renoprotective effects of miR-27a inhibition in diabetic rats.

## Discussion

Mounting evidence has implicated miRNAs in the development of DN. For instance, miR-192 levels are significantly increased in diabetic glomeruli, and this miRNA influences TGF-β-induced collagen 1-α2 expression by downregulating E-box repressors^36^. In addition, downregulation of miR-29c reduces podocyte apoptosis and decreases ECM protein accumulation, both *in vitro* and *in vivo*^37^. miR-377 is upregulated in human and mouse MCs exposed to HG and indirectly stimulates increased fibronectin protein production^38^. While miR-27a is reportedly upregulated in the serum of patients with type 1 or 2 diabetes^11^,^12^, there have been no reports of pathological roles of miR-27a in DN to date. Here, we found that miR-27a expression was consistently upregulated in rat MCs exposed to HG and in kidney glomeruli from STZ-induced diabetic rats. Our results also indicated that miR-27a was capable of targeting the PPARγ 3′-UTR to inhibit PPARγ expression. Further, miR-27a upregulation led to enhanced cell proliferation and ECM accumulation through PPARγ downregulation. Moreover, the in vivo inhibition of miR-27a protected diabetic renal function and ameliorated the progression of DN. Therefore, miR-27a is likely to be relevant in DN pathology, and is potentially an important therapeutic target for treating diabetic renal diseases.

Emerging evidence has suggested the importance of miR-27a overexpression in diabetes^11^,^12^,^13^, although the full molecular mechanisms remain to be established. miR-27a is upregulated in cultured adipocytes exposed to hyperglycaemia^13^. Moreover, miR-27a levels are strongly and positively correlated with fasting glucose levels in patients with type 2 diabetes^12^. Together, these findings indicate that increased miR-27a expression may be involved in the initial cellular responses to hyperglycaemia, which could eventually result in the development of diabetes. To determine whether miR-27a is a key regulatory factor in DN, we examined its levels in samples obtained from kidney glomeruli of STZ-induced diabetic rats and in kidney MCs exposed to hyperglycaemic conditions. Our integrated *in vitro* and *in vivo* studies indicated that miR-27a expression was significantly increased in these tissues and cell types. Importantly, inhibition of miR-27a with a miR-27a inhibitor *in vitro* or with antagomir-27a *in vivo* prevented HG-induced MC proliferation and ECM accumulation. Furthermore, antagomir-27a treatment significantly ameliorated proteinuria and renal pathology in STZ-induced diabetic rats. Although the regulatory influences of miR-27a remain incompletely defined, our data reveal that its overexpression clearly affects DN development.

Growing evidence indicates that PPARγ activation is associated with the attenuation of DN. Our data showed that PPARγ expression was decreased in kidney MCs exposed to hyperglycaemic conditions, which is in agreement with data in previous reports^20^,^29^. Interestingly, the expression of miR-27a increased while PPARγ expression decreased in HG-treated MCs. Recently, it was reported that miR-27a could directly bind to the 3′-UTR of PPARγ mRNA and suppress PPARγ expression, thereby suppressing adipocyte differentiation^14^. Similarly, miR-27a can bind to the PPARγ 3′-UTR in human pulmonary artery endothelial cells and regulate cell proliferation^17^. Therefore, we hypothesized that increased miR-27a levels may provide the mechanistic explanation for reduced PPARγ expression. We indeed confirmed that miR-27a bound to the PPARγ 3′-UTR in rat kidney MCs. Furthermore, our results demonstrated that a miR-27a mimic significantly reduced PPARγ expression in NG-treated MCs. Interestingly, we observed increased PPARγ expression in the kidney glomeruli of diabetic rats, which was comparable to the upregulation seen in a study by Nicholas *et al*.^32^. PPARγ upregulation may serve to limit injury responses in the kidneys of individuals with diabetes^32^. However, the increase in PPARγ expression was insufficient to inhibit diabetic renal injury. Importantly, we showed that the knockdown of miR-27a with antagomir-27a significantly increased PPARγ expression. Taken together, these results highlight miR-27a as an important regulator of PPARγ expression in DN.

The pathological hallmark of diabetic glomerular lesions is ECM accumulation^2^,^39^, and activation of PPARγ can reduce MC proliferation and suppress ECM accumulation via inhibition of TGF-β1 responses^3^, and by reducing PAI-1 expression^32^. Consistent with previous studies, our studies demonstrated that PPARγ downregulation induced MC proliferation and increased levels of profibrotic genes in MCs exposed to hyperglycaemic conditions. In addition, the PPARγ upregulation observed following miR-27a knockdown was associated with decreased MC proliferation and reduced expression of profibrotic genes. Importantly, knockdown of PPARγ abrogated the effects of miR-27a inhibitor on MC proliferation and profibrotic gene expression, indicating the critical role of PPARγ as a target of miR-27a in both HG-induced cell proliferation and ECM accumulation in rat kidney MCs. Consistent with these *in vitro* results, there was a decrease in miR-27a expression and a corresponding increase in PPARγ expression in antagomir-27a-treated diabetic rats; this reduced cell proliferation, and inhibited ECM accumulation compared with untreated diabetic rats and negative control rats. These data indicate that miR-27a negatively regulates PPARγ, and in doing so, triggers enhanced MC proliferation and ECM accumulation.

Taken together, the data presented in this study reveal for the first time the critical role of miR-27a in the response of MCs exposed to HG concentrations. In addition, we show that miR-27a is a novel regulator of MC proliferation and ECM accumulation through the post-transcriptional regulation of PPARγ. Furthermore, our data suggest that antagomir-based miRNA inhibitors (such as antagomir-miR-27a) can efficiently and specifically reduce renal miR-27a levels, renal hypertrophy, profibrotic gene expression, and renal fibrosis in diabetic rats. These findings provide new insights into the role of miR-27a in diabetes and indicate that miR-27a inhibitors may be useful for the treatment of DN.

## Additional Information

**How to cite this article**: Wu, L. *et al*. MicroRNA-27a Induces Mesangial Cell Injury by Targeting of PPARγ, and its In Vivo Knockdown Prevents Progression of Diabetic Nephropathy. *Sci. Rep.* **6**, 26072; doi: 10.1038/srep26072 (2016).
